# Moving towards personalized medicine in rheumatoid arthritis

**DOI:** 10.1186/ar4565

**Published:** 2014-05-19

**Authors:** Tamarah D de Jong, Saskia Vosslamber, Cornelis L Verweij

**Affiliations:** 1 Department of Pathology, Section of Inflammatory Disease Profiling, VU University Medical Center, Boelelaan 1118, 1081 HZ, Amsterdam, The Netherlands; 2 Department of Rheumatology, Section of Inflammatory Disease Profiling, VU University Medical Center, Boelelaan 1117, 1081 HV, Amsterdam, The Netherlands

## Abstract

To develop personalized medicine strategies for improvement of patient management in rheumatoid arthritis, the clinical and molecular properties of the individual patients need to be well characterized. A crucial step in this approach is to discover subgroups of patients that are characterized by a good or poor treatment outcome. Dennis and colleagues have identified distinct pretreatment gene expression profiles in affected synovial tissue specimens and a tissue type-related systemic protein pattern which are associated with a positive or negative clinical outcome to monotherapy with adalumimab (anti-TNFα) and tocilizumab (anti-IL-6 receptor). These observations assign biological pathways associated with response outcome and provide evidence for the existence of systemic, easy-to-measure predictive biomarkers for clinical benefit of these biologics.

## 

In rheumatoid arthritis (RA), therapies with biologics targeting TNFα, IL-6 receptor, T-cell co-stimulation, and B cells are successfully used worldwide. Since not all patients benefit from these specific therapies, there is a strong unmet need for pretreatment predictions on therapy outcome. This concept of personalizing treatment has raised interest in the discovery of clinically applicable pretreatment biomarkers to make predictions on outcome before the start of treatment. In this issue of *Arthritis Research & Therapy*, Dennis and colleagues [1] report on two tissue type-related blood-based protein biomarkers that are associated with the clinical response to adalimumab (anti-TNFα) and tocilizumab (anti-IL-6 receptor).

Global gene expression profiling is a powerful method for biomarker discovery purposes and has proven to be useful in the identification of potentially useful biomarkers in cancer [2,3]. In RA, this approach yielded a potentially useful blood-based biomarker for the prediction of outcome of B-cell depletion therapy using rituximab [4]. However, very inconsistent results were obtained for TNF blockers, raising skepticism about the clinical utility of the reported gene signatures [5,6]. These controversial results for TNF blockers may indicate the complex interplay between pathogenesis and anti-TNF pharmacology in RA.

Dennis and colleagues [1] describe a multistep process to identify predictive biomarkers in serum for adalimumab and tocilizumab. Their study is based on the premise of the existence of different molecular synovial phenotypes between patients with RA, as has been noted before [7]. Firstly, the authors identified four clearly distinct molecular synovial phenotypes by using global expression profiling. Gene ontology analysis indicated the existence of two inflammatory axes: one with a lymphoid phenotype, characterized by B-cell abundance, and another with a myeloid phenotype, characterized by activated M1-monocyte enrichment. The remaining two subtypes represented a low inflammatory phenotype and a fibroblast activation phenotype.

Secondly, only the myeloid phenotype appeared to be associated with European League Against Rheumatism good-versus-poor response to the TNF blocker infliximab. Receiver operating characteristic (ROC) analysis revealed an area under the curve (AUC) of 77% for the myeloid-associated gene set, indicating that pretreatment transcript levels of the synovial myeloid phenotype could potentially enrich for good responders to TNF blockers.

Thirdly, to translate these findings to a more easily accessible compartment, the authors focused on two genes, C-X-C motif chemokine 13 (*CXCL13*) and the intercellular adhesion molecule 1 (*ICAM1*), enriched in the lymphoid and myeloid synovial phenotypes, respectively, each encoding a soluble product that could serve as a systemic biomarker. Subsequent measurements of CXCL13 and soluble ICAM1 (sICAM1) in pretreatment serum samples from the ADACTA (ADalimumab ACTemrA) trial (comparing adalimumab with tocilizumab) revealed that CXCL13^low^/sICAM1^high^ patients had the highest American College of Rheumatology (ACR)-based clinical responses to adalimumab after 24 weeks, whereas CXCL13^high^/sICAM1^low^ patients had the highest responses to tocilizumab. ROC AUC values for the individual biomarkers reached 65%, based on ACR 50% improvement criteria response outcome.

The scientific value of these findings comes from the identification of two distinct highly inflammatory synovial tissue subtypes that appear to be key to identifying anti-TNFα and anti-IL-6 receptor responders. This corroborates findings from others of a correlation between baseline macrophages and TNFα levels with clinical response [8,9]. Successful action of TNFα blockers may rely on the presence of a myeloid phenotype representing inflammatory M1 monocytes, which may constitute a key lineage in TNFα-activated nuclear factor-kappa-B-driven synovitis. The TNFα-regulated and membrane-shed ICAM1 appears to be an exponent of the myeloid tissue phenotype. In contrast, the B-cell dominant highly inflammatory lymphoid phenotype appears to be selectively driven by the IL-6/IL-6 receptor pathway and its JAK/STAT-associated transcription factor STAT3. Surprisingly, this phenotype was not characterized by selective expression of IL-6 or IL-6-related genes (*IL-6R*, *IL-6ST/gp130*, and *STAT3*), indicative of the pleiotropic role of IL-6 in different processes of human biology associated with different tissue subsets. Synovium-derived circulating CXCL13 as a B-cell chemoattractant could explain B-cell trafficking into lymphoid phenotype tissues. Since CXCL13 is selectively expressed by follicular dendritic cells, it remains to be established whether the lymphoid phenotype is associated with the presence of ectopic germinal centers.

Although the results of Dennis and colleagues [1] are scientifically important and demonstrate the importance of gene expression profiling in the search for predictive biomarkers, the individual patient-based ROC analyses show only modest predictive ability and therefore weak clinical utility. As mentioned by the authors, the problem for the weak performance may lie in the linear rather than discrete distribution of the synovial phenotypes. Therefore, further refinement and incorporation of additional biomarkers may improve the clinical utility.

All together, these results indicate that molecular phenotyping of patients with RA clearly contributes to the development of a personalized medicine approach for TNFα and IL-6 blockade. Moreover, detailed analysis of specific patterns may lead to new therapeutic avenues. Usage of predictive biomarkers in combination with those for other biologics, such as rituximab, might lead to an early-triage approach for enrichment of those patients most likely to respond to a distinct biologic in RA.

## Abbreviations

ACR: American College of Rheumatology; AUC: Area under the curve; CXCL13: C-X-C motif chemokine 13; ICAM1: Intercellular adhesion molecule 1; IL: Interleukin; RA: Rheumatoid arthritis; ROC: Receiver operating characteristic; sICAM1: Soluble intercellular adhesion molecule 1; TNF: Tumor necrosis factor.

## Competing interests

CLV is an inventor on a patent in which the prediction of the response to rituximab is claimed, and he is a stakeholder in Preselect Diagnostics BV (Abcoude, The Netherlands). The other authors declare that they have no competing interests.
